# Genome-Wide Analysis of Serine Hydroxymethyltransferase Genes in Triticeae Species Reveals That *TaSHMT3A-1* Regulates Fusarium Head Blight Resistance in Wheat

**DOI:** 10.3389/fpls.2022.847087

**Published:** 2022-02-10

**Authors:** Ping Hu, Puwen Song, Jun Xu, Qichao Wei, Ye Tao, Yueming Ren, Yongang Yu, Dongxiao Li, Haiyan Hu, Chengwei Li

**Affiliations:** ^1^Henan Engineering Research Center of Crop Genome Editing, Henan International Joint Laboratory of Plant Genetic Improvement and Soil Remediation, College of Life Science and Technology, Henan Institute of Science and Technology, Xinxiang, China; ^2^School of Horticulture and Landscape Architecture, Henan Institute of Science and Technology, Xinxiang, China; ^3^Department of Plant Protection, Sumy National Agrarian University, Sumy, Ukraine; ^4^College of Biological Engineering, Henan University of Technology, Zhengzhou, China

**Keywords:** genome-wide analysis, SHMT gene family, expression pattern, Fusarium head blight, virus-induced gene silencing, evolutionary progress

## Abstract

Serine hydroxymethyltransferase (*SHMT*) plays a pivotal role in cellular one-carbon, photorespiration pathways and it influences the resistance to biotic and abiotic stresses. However, the function of SHMT proteins in wheat remains largely unexplored. In the present study, *SHMT* genes in five *Triticeae* species, *Oryza sativa*, and four dicotyledon species were identified based on whole genome information. The origin history of the target gene was traced by micro-collinearity analysis. Gene expression patterns of *TaSHMTs* in different tissues, various biotic stresses, exogenous hormones, and two biotic stresses were determined by Quantitative real-time reverse transcription polymerase chain reaction (qRT-PCR). The function of the selected *TaSHMT3A-1* was studied by barley stripe mosaic virus-induced gene silencing in common wheat Bainong207. A total of 64 *SHMT* members were identified and further classified into two main classes based on the structure of SHMT proteins. The gene structure and motif composition analyses revealed that *SHMTs* kept relatively conserved within the same subclasses. Interestingly, there was a gene, *TdSHMT7B-1*, on chromosome 7B of *Triticum dicoccoides*, but there was no *SHMT* gene on chromosome 7 of other analyzed *Triticeae* species; *TdSHMT7B-1* had fewer exons and conserved motifs than the genes in the same subclass, suggesting that the gene of *TdSHMT7B-1* has a notable evolutionary progress. The micro-collinearity relationship showed that no homologs of *TaSHMT3A-1* and its two neighboring genes were found in the collinearity region of *Triticum urartu*, and there were 27 genes inserted into the collinearity region of *T*. *urartu*. Furthermore, qRT-PCR results showed that *TaSHMT3A-1* was responsive to abiotic stresses (NaCl and cold), abscisic acid, methyl jasmonate, and hydrogen peroxide. Significantly, upon *Fusarium graminearum* infection, the expression of *TaSHMT3A-1* was highly upregulated in resistant cultivar Sumai3. More importantly, silencing of *TaSHMT3A-1* compromises Fusarium head blight resistance in common wheat Bainong207. Our new findings suggest that the *TaSHMT3A-1* gene in wheat plays an important role in resistance to Fusarium head blight. This provides a valuable reference for further study on the function of this gene family.

## HIGHLIGHTS

- 64 *SHMT* genes were systematically analyzed in 10 species
- Wheat *TaSHMT*’ evolution was analyzed with micro-collinearity analysis
- Some *TaSHMTs* responded to abiotic, biotic, and hormone treatments
- *TaSHMT3A-1* in wheat plays an important role in resistance to Fusarium head blight

## Introduction

Wheat (*Triticum aestivum*) is one of the most important staple crops in the world, and is a heterologous hexaploid composed of three subgenomes of A, B, and D (IWGSC, 2018). Wheat is subjected to various biotic and abiotic stresses throughout its life cycle. Fusarium head blight (FHB) and powdery mildew seriously affect the yield and quality of wheat (Hu et al., 2018; Wang et al., 2020). Deoxynivalenol (DON) mycotoxins, produced by *Fusarium graminearum* in infected grains seriously affect the safety of human food and animal feed (Li et al., 2019; Wang et al., 2020). Changes in climate and crop planting systems have made FHB increasingly serious, even in regions where it has not been reported before (Del Ponte et al., 2009; Chakraborty and Newton, 2011; McMullen et al., 2012; Zhang et al., 2014). Mining disease-resistant genes and cultivating disease resistant varieties are the most economical and effective strategies to reduce the losses caused by disease (Bai and Shaner, 2004; Dean et al., 2012; Xing et al., 2018).

Serine hydroxymethyltransferase (SHMT), a pyridoxal phosphate-dependent enzyme, can catalyze the glycine/serine and tetrahydrofolate (THF)/5,10-methyleneTHF interconversion and it plays a vital role in one-carbon metabolism and photorespiration Gly-into-Ser conversion in higher plants (Schirch and Szebenyi, 2005; Lakhssassi et al., 2019). The *SHMT* gene family is widely present in the form of dimer in prokaryotes and tetramer in eukaryotes (Prabhu et al., 1996). In humans and animals, *SHMTs* are related to multiple diseases, including cancer and ischemic stroke (Garcia-Canaveras et al., 2021). There has been considerable investigation of the *SHMT* gene in animals and humans, but relatively few studies in plants. In plants, previous studies have identified SHMT activity in different intracellular compartments, including mitochondria, plastids, cytosol, and nuclei (Turner et al., 1992; Neuburger et al., 1996; Zhang et al., 2010; Lakhssassi et al., 2019). Seven SHMT genes in *Arabidopsis* (McClung et al., 2000), five in *Oryza sativa* (Ohyanagi et al., 2006), and 18 in soybean (Lakhssassi et al., 2019) have been identified. In *Arabidopsis*, *AtSHM1* is involved in the photorespiratory pathway (McClung et al., 2000; Moreno et al., 2005; Voll et al., 2006). The mutant *shm1-1* showed a lethal photorespiratory phenotype caused by photorespiration deficiency when grown at ambient CO_2_ (Voll et al., 2006), and showed a more susceptible phenotype than the wild type when infected with biotrophic and necrotrophic pathogens, as well as enhanced susceptibility to salt, drought, and high light stress (Moreno et al., 2005; Liu et al., 2019; Mishra et al., 2019). *AtSHM2* is a functional mitochondrial *SHM*, and the expression of *SHM2* is restricted to the vasculature of leaves (Engel et al., 2011). *AtSHM2* and *AtSHM1* operate synergistically in photorespiration, but *AtSHM2* cannot substitute for *AtSHM1* in photorespiratory metabolism. Overexpression of *SHM2* cannot complement the *shm1* allele, although the amino acid sequences of SHM1 and SHM2 are very similar (Voll et al., 2006; Engel et al., 2011). AtSHMT3 is targeted to plastids, and biochemical experiments show that SHMT activity in plastids of both *Arabidopsis* and *Hordeum vulgare*, and *AtSHMT3* is also involved in one-carbon metabolism in plants (Zhang et al., 2010). In rice, *OsSHMT1* is an ortholog of *AtSHM1*, and has been identified in photorespiratory mutant *osshm1* (Wang et al., 2015). Studies indicate a conserved function of *SHMT1* in photorespiration in rice and *Arabidopsis*. Rice *SHMT3* confers tolerance to salinity stress in heterologous *Arabidopsis* (Mishra et al., 2019). Overexpression of the halotolerant cyanobacteria *Aphanothece halophytica* gene *ApSHMT* in *Escherichia* coli induced an enhanced tolerance to salinity-stress (Waditee-Sirisattha et al., 2012). The expression level of wheat *SHMT* was significantly reduced when wheat plants were exposed to soil drought and PEG-induced stresses (Cui et al., 2019). In soybean, the *GmSHMT* gene showed a lack of functional redundancy in resistance to soybean cyst nematode (Liu et al., 2012; Kandoth et al., 2017; Lakhssassi et al., 2019). These studies suggest that *SHMT* genes are not only involved in photorespiration but also salt, drought, and disease resistance in different plant species.

In previous research, most investigations of the *SHMT* gene have focused on the photorespiration of dicotyledons, and there have been only a few studies on the systematic analysis of the *SHMT* gene family in monocotyledons, especially in biotic stress of *Triticeae* species. Based on the whole genome information of wheat and its related species, this study defined the gene structure and evolutionary relationship of *SHMT* genes in *Triticeae* species and three dicotyledonous horticultural crops. Quantitative real-time reverse transcription polymerase chain reaction (qRT-PCR) was used to systematically analyze the expression pattern of *SHMT* genes in different wheat varieties under biotic and abiotic stresses, and hormone treatment, such as *F. graminearum*, powdery mildew, drought, NaCl, cold (4°C), abscisic acid (ABA) and hydrogen peroxide (H_2_O_2_). To further verify the function of *TaSHMT3A-1*, which is in response to *F. graminearum* infection in Sumai3, the technology of barley stripe mosaic virus-induced gene silencing (BSMV- VIGS) was used to verify its function in wheat, and the results showed that silencing of *TaSHMT3A-1* could increase the susceptibility of Bainong207 to FHB. This study will provide a foundation for further functional studies of the *SHMT* gene family.

## Materials and Methods

### Plant Materials and Growth Conditions

The common wheat Chinese cultivar Sumai3 which carries *Fhb1* and has consistently shown a major effect on resistance to FHB (Li et al., 2019; Su et al., 2019) was collected and maintained by Henan Institute of Science and Technology (HIST), Xinxiang China. FHB susceptible common wheat Jimai22 was collected from the Shandong Academy of Agricultural Sciences, and preserved by HIST. Bainong207 was developed and maintained by HIST. Sumai3, Jimai22, and Bainong207 were used for gene expression analysis and leaves from three different individuals of the same treatment were collected and mixed at each time point. Bainong207 was also used for the BSMV-VIGS assay. Tissue expression of *TaSHMTs* in root, stem, and leaf were examined at the adult stage of Sumai3. For the abiotic stress treatments, 14-day-old wheat seedlings of Bainong207 were treated with 20% PEG6000, 200 mmol NaCl, 4°C, 100 μmol methyl jasmonate (MeJA), 100 μmol H_2_O_2_, and 100 μmol ABA. Leaf samples were collected after 1, 12, and 24 h treatment. Bainong207 was used for expression analysis and grown in a climatic chamber at 23°C/18°C, with a 14 h light/10 h dark cycle, and 70% relative humidity.

### *Blumeria graminis* f. sp. *tritici*, *Fusarium graminearum* Preparation and Plants Treatments

Mixed races of *Blumeria graminis* f. sp. *tritici (Bgt)* were collected in fields in Xinxiang, China and preserved on seedlings of the high susceptible variety Sumai3 in the climatic chamber. For RNA extraction, total RNAs were extracted from seedling leaves of Bainong207 inoculated with *Bgt* at 0, 6, 24, 48, and 72 hpi (hours post-inoculation) using Trizol reagent (Vazyme, Nanjing, China) following the manufacturer’s protocol. The *F. graminearum* used in this study was a field isolate originating in Henan, China and preserved by HIST. The spikelets of Sumai 3 and Jimai 22 were inoculated at the early flowering stage with 20 μL fresh spores of *F. graminearum* in the middle of the heads by the single-floret inoculation method. The spore concentration was 10^1^ conidia mL^–1^. Three spikelets (from three inoculated spikes of different individuals) were collected at 0, 24, 36, 48, and 72 hpi. All these three materials of Bainong207, Sumai3, and Jimai22 were grown in a greenhouse at 23°C/18°C, with a 14 h light/10 h dark cycle, and 70% relative humidity.

### Expression Analysis of *TaSHMTs* by Quantitative Real-Time Reverse Transcription Polymerase Chain Reaction

The qRT-PCR procedure was performed as described by Hu et al. (2018). The first-strand cDNA was synthesized using the HiScript Q RT SuperMix for qRT-PCR Kit (Vazyme, Nanjing, China). The qRT-PCR was performed using the SYBR Green detection kit AceQ qPCR SYBR Green Master Mix (Vazyme, Nanjing, China) on LC 480II (Roche, German). The program used was as follows: 5 min at 95°C, followed by 40 cycles at 95°C for 10 s and 60°C for 20 s. The relative gene expression was calculated by comparative 2^–ΔΔCT^ method. The primers used in this study are listed in Supplementary Table 1 and the wheat *TaTubulin* gene was used as an internal control.

### Identification of *SHMT* Gene

The genome-wide data for *T. aestivum* (Chinese Spring) from IWGSC^2^ was downloaded (IWGSC, 2018). Data for *Triticum urartu* (Tu 2.0) were downloaded from the MBKBase database^3^ (Ling et al., 2018). *Triticum dicoccoides* (WEWSeq_v.1.0), *Aegilops tauschii* (Aet_v4.0), *Hordeum vulgare* (IBSC_v2), *Arabidopsis thaliana* (TAIR10), *Solanum lycopersicum* (SL3.0), *Cucumis sativus* (ASM407v2), and *Vitis vinifera* (12X) were downloaded from the Ensemble Plants^4^ to construct a local database. The typical SHMT domain (PF00464) was downloaded from the Pfam database as the search model^5^ (El-Gebali et al., 2019). As described in Xu et al. (2021), a new hidden Markov model (HMM) was established to ensure the reliability of search results. The high-quality protein set was obtained using the raw SHMT HMM (*E*-value < 1 × 10 ^–20^ and manual validation of an intact SHMT domain), and then used to build a specific SHMT HMM by the hmmbuild from the HMMERv3 suite (Lozano et al., 2015). The specific HMM was used, and proteins with an *E*-value lower than 0.001 were retained. The longest transcript was included for the following analysis when a gene has multiple transcripts. Both The SMART (Simple Modular Architecture Research Tool)^5^ (Letunic et al., 2021) and Conserved Domains^6^ (Lu et al., 2020) were used to check the candidate SHMT protein sequences again. The proteins containing complete SHMT conserved domains were reserved for further analysis, and named sequentially to their species and location on the chromosomes; all gene names are listed in Supplementary Table 2.

### Phylogenetic, Gene Structure, and Conserved Motif Analysis

Multiple sequence alignment of all these full-length SHMT proteins was performed with ClustalW using the default options in MEGA-X (Kumar et al., 2018). Phylogenetic trees were constructed using the Maximum likelihood method of MEGAX with 1000 bootstrap replicates (Kumar et al., 2018; Yu et al., 2020). The phylogenetic tree was visualized with EvolView^7^ (He et al., 2016). The exon-intron structure was generated using TBtools based on the full-length genome sequence and the corresponding coding sequences (Chen C. et al., 2020). Conserved motifs analysis was performed using the MEME program^8^. The parameters were as follows: the maximum number of motifs was set to 20 and the optimum width was 6–50 residues (Xie et al., 2018; Xu et al., 2021). The gene structure with motif composition was visualized by the TBtools (Chen C. et al., 2020).

### Micro-Collinearity and Functional Diversification Analysis

A micro-collinearity analysis is of great value for understanding gene evolutionary history and TGT (Triticeae-Gene Tribe^9^) was used to trace the origin history of the target gene, and gene pair was also analyzed with TGT (Chen Y. et al., 2020). DIVERGE v3.0 software was used to analyze the functional diversification among the subgroups based on the selected protein sequences (Gu et al., 2013).

### BSMV-VIGS

BSMV-VIGS was performed as described by Wang et al. (2010) with some modifications. The fragment of *TaSHMT3A-1* with the length of 249 bp was amplified with primer pair VIGS-*TaSHMT3A-1* (Supplementary Table 1), and the target fragment was inserted into the γ-strain of BSMV to construct BSMV: *TaSHMT3A-1* vector. An *in vitro* transcription kit (mMESSAGEmMACHINE T7, Invitrogen, Waltham, MA, United States) was used to produce the virus RNA. The common wheat Bainong207 was used for the gene silencing assay. When the second leaves were fully extended, the leaves infected with the virus BSMV: *TaSHMT3A-1*, with BSMV*:TaPDS*- and BSMV:γ- infected leaves as controls. The fourth leaves fully expanded with clear virus symptoms were used for disease resistance evaluation, and the inoculation method was performed as described in Xiang et al. (2011) with some modifications. The fourth leaves were detached from Bainong207, wounded on the adaxial surface, and then placed on the PCR board to form arch bridges. For *F. graminearum* inoculation, the inoculum comprised 1.5 μL of conidial suspension with a concentration at 1 × 10^6^ conidia mL^–1^. The conidial suspension of *F. graminearum* was applied to the fresh wound, and then the PCR board with leaves was placed in 25 mgL^–1^ benzimidazole water, at the same time ensuring that the cut of the leaves contacted the water, and then sealed for moisturizing. The inoculated leaves were cultured in an incubator with 14 h light/8 h dark at 23°C. The lesion size was observed 3 and 5 days after inoculation. Target gene silencing efficiency was evaluated by qRT-PCR using the primer pair *TaSHMT3A-1*-Q (Supplementary Table 1).

## Results

### Identification and Classification of *SHMT* Gene Family Members in Triticeae and Four Dicotyledon Species

**FIGURE 1:**
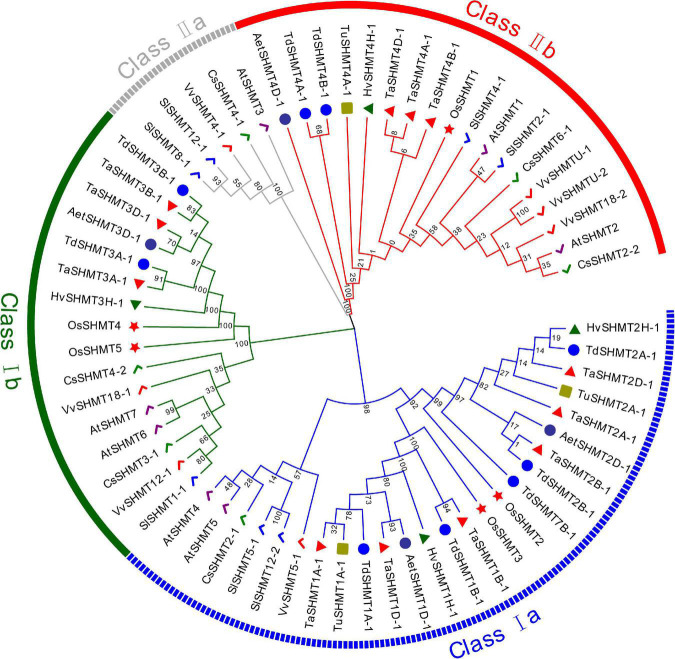
Phylogenetic relationship analysis of 64 SHMT proteins from *T. aestivum*, *T. urartu*, *Ae. Tauschii*, *T. dicoccoides*, *H. vulgare*, *O. sativa*, *Arabidopsis*, *S. lycopersicum, C. sativus*, and *V. vinifera.* The phylogenetic tree was built using the Maximum likelihood method (ML) with 1000 bootstrap replicates by MEGA X. The diverse subgroups of SHMT proteins were marked with different colors. The SHMT proteins of *T. aestivum*, *T. urartu*, *Ae. Tauschii*, *T. dicoccoides*, *H. vulgare*, *O. sativa*, *Arabidopsis*, *S. lycopersicum, C. sativus*, and *V. vinifera* were represented by red triangles, green squares, purple circles, blue circles, green triangles, red stars, purple checkmarks, blue checkmarks, green checkmarks, and red checkmarks, respectively. Gene IDs of the analyzed genes can be found in Supplementary Table 2.

In this study, a total of 64 genes with SHMT conserved domain (PF00464) were identified from 10 sequenced plant species, 37 from monocotyledon, including *T. aestivum* (12), *T. urartu* (3), *Ae. Tauschii* (4), *T. dicoccoides* (9), *H. vulgare* (4) and *O. sativa* (5), and 27 from dicotyledons, including *Arabidopsis* (7), *S. lycopersicum* (7), *C. sativus* (6), and *V. vinifera* (7). To investigate the evolutionary relationships of the SHMT proteins, all the above 64 proteins were used to construct a Maximum likelihood phylogenetic tree (Figure 1). According to the classification of *Arabidopsis* and soybean SHMT (Lakhssassi et al., 2019), SHMT is divided into two main classes (classes I and II) and four subclasses (classes I a–b, II a, and II b). Among them, Class II a subgroup only contains proteins from dicotyledons, and the other subgroups include proteins from monocotyledons and dicotyledons. Within the same subgroup, the *SHMT* members of monocotyledons and dicotyledons were clustered together respectively, indicating that the *SHMT* genes of monocotyledons and dicotyledons had experienced great differentiation in the process of evolution. SHMT members within the same subgroup of *Triticeae* species had high protein sequence similarity, and the evolutionary process in *Triticeae* species was relatively conservative.

The numbers of *SHMT* in each subgroup of all analyzed species are listed in Table 1. Compared with monocotyledons, dicotyledons have more SHMT genes, mainly reflected in class I b, class II a, and class II b subgroups (Table 1). In Triticeae species, *T. dicoccoides* and *T. aestivum* had 9 and 12 *SHMTs*, which was about two and three times that of diploid species, respectively; moreover, the number of *SHMTs* in each subgroup was almost 2–3 times that of the diploid species (Table 1). This indicated that the increased number of *TaSHMTs* in polyploid wheat was primarily due to genome polyploidization. Interestingly, there was no *SHMT* gene of *T. urartu* in class I b, and the *SHMT* genes of barley and *Ae. Tauschii* were distributed on chromosomes 1, 2, 3, and 4, while *T. urartu* lacked the *SHMT* gene on chromosome 3; moreover, the *SHMT* genes on chromosome 3 of *Ae. tauschii* and barley were classed in class I b (Figure 1 and Table 2). This may be due to either a poor reference genome sequence of *T. urartu* or gene loss events that occurred during the evolution of *T. urartu.* The proportion of *SHMT* gene and copy number in class I a of *T. dicoccoides* was higher than that of other *Triticeae* species and there was an *SHMT* gene, *TdSHMT7B-1*, on chromosome 7B, but there was no *SHMT* gene on chromosome 7 of other *Triticeae* species. From the perspective of evolutionary relationships, the number and evolution of *SHMT* genes have differentiated between monocotyledons and dicotyledons, and within *Triticeae* species. Therefore, gene structure and conserved motifs of SHMT in different species were further analyzed to help explore the evolutionary process.

**TABLE 1:** Numbers of *SHMT* homologs encoded by the surveyed genomes in total and individual subclasses.

Genome	Total number	Subgroup			
		Class Ia	Class Ib	Class IIa	Class IIb
*H. vulgare* (HH)	4	2	1	0	1
*T. urartu* (AA)	3	2	0	0	1
*Ae. Tauschii* (DD)	4	2	1	0	1
*T. dicoccoides* (AABB)	5 (9)	3 (5)	1 (2)	0	1 (2)
*T. aestivum* (AABBDD)	4 (12)	2 (6)	1 (3)	0	1 (3)
*O. sativa*	5	2	2	0	1
*A. thaliana*	7	2	2	1	2
*V. vinifera*	7	1	2	1	3
*S. lycopersicum*	7	2	1	2	2
*C. sativus*	6	1	2	1	2
Total	52 (64)	19 (25)	13 (16)	5	15 (18)

*Numbers in brackets indicate number of copies for polyploid genomes.*

**TABLE 2:** Number of *SHMT* from different Triticeae species in each of the chromosomes.

Chromosome	*T. aestivum*			*T. dicoccoides*		*T. urartu*	*Ae. tauschii*	*H. vulgare*	Total
	A	B	D	A	B	A	D	H	
Chr.1	1	1	1	1	1	1	1	1	8
Chr.2	1	1	1	1	1	1	1	1	8
Chr.3	1	1	1	1	1	0	1	1	7
Chr.4	1	1	1	1	1	1	1	1	8
Chr.5	0	0	0	0	0	0	0	0	0
Chr.6	0	0	0	0	0	0	0	0	0
Chr.7	0	0	0	0	1	0	0	0	1
Total	4	4	4	4	5	3	4	4	32
									

### Gene Structure and Conserved Motif Composition Analysis

**FIGURE 2:**
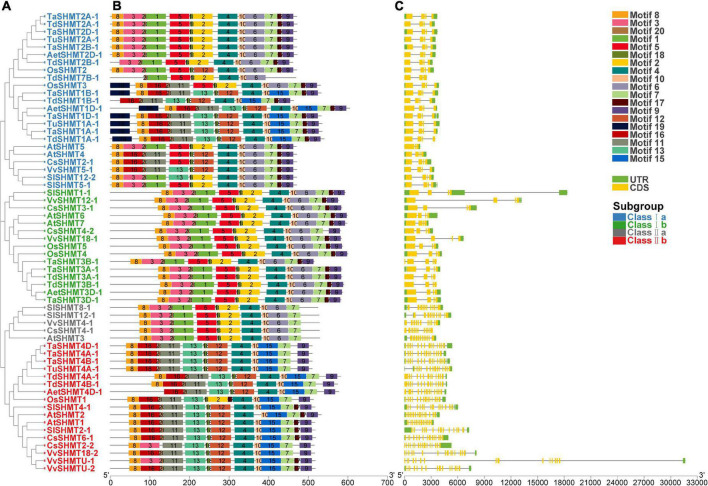
Phylogenetic relationships, conserved motifs, and gene structure of *SHMT* genes in Triticeae species. **(A)** The phylogenetic tree was constructed using the neighbor-joining method with 1,000 bootstrap replicates by MEGA X. **(B)** The motif composition of SHMT proteins. The motif compositions of SHMT proteins. The 20 motifs were indicated by colored boxes and numbered 1–20. **(C)** Exon–intron structure of *SHMTs*. Yellow boxes indicated exons; black lines indicated introns.

To further understand SHMT functional divergence, the conserved motifs of these SHMT proteins were identified using MEME software. Twenty individual motifs were identified (Figure 2). Our results show that, among all the analyzed 60 SHMT, proteins contained motif 4, motif 7 (except for TdSHMT7B-1), motif 10, and motif 18. All the analyzed SHMTs in Class I contained motif 9 (except for TdSHMT7B-1) and motif 17 (except for TdSHMT7B-1 and CsSHMT4-2); however, motif 9 and motif 17 lacked in the protein sequence of class II a (Figures 2A,B). Exon–intron structure divergence plays an important role during the evolution of duplicate genes and different compositions of the motif are important for their functional diversity (Yu et al., 2020; Xu et al., 2021). The intron-exon structure was analyzed by the aligning the full-length cDNA with genomic DNA sequence (Figure 2). The structure analysis of *SHMT* genes indicated that closely related members had a similar exon-intron structure. In class I, the number of exons ranged from three to five; among the 38 analyzed *SHMT* genes, 35 genes had four exons, and only *TdSHMT7B-1* and *AtSHMT4* in class I a had three exons, *TaSHMT3B-1* in class I b had five exons (Figures 2A,C). Compared with class I, there were great differences in the intron–exon structure between class I and class II, each *SHMT* gene in class II had multiple short exons. Class II a, which is only composed of dicotyledon genes, contained 11 exons. Class II b contained 17 analyzed genes in total, and the number of exons from 13 to 16, especially, *TdSHMT4A-1*, had 13 exons. *TdSHMT4B-1* and *AetSHMT4D-1* had 14 exons, *CsSHMT2-2* had 16 exons, and all the other 13 genes had 15 exons (76.47%). These data suggest that the exon-intron structure and motifs in SHMT were highly correlated with phylogenetic relationships and special motifs in SHMT may play critical roles in specific functions.

### Micro-Collinearity Analysis of Triticeae

**FIGURE 3:**
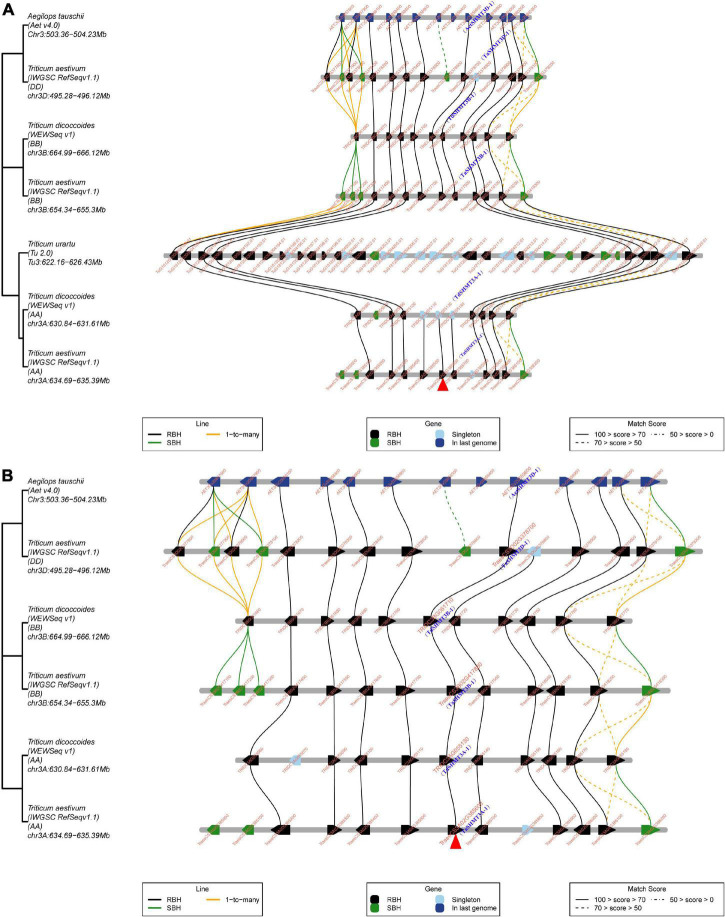
Micro-collinearity analysis by TGT to track the evolutionary history of *TaSHMT3A-1* gene homologs. **(A,B)** *TaSHMT3A-1* was used as the query gene. The micro-collinearity relationship showed that no homolog of *TaSHMT3A-1* and its two neighboring genes was found in the collinearity region of *T. urartu*, but other genes were relatively conserved across other investigated genomes and there were 27 genes inserted into the collinearity region of *T. urartu*. The red arrow indicates *TaSHMT3A-1* **(A)**. In the micro-collinearity relationship analyze the genome of *T. urartu* was deleted. The neighboring genes of *TaSHMT3A-1* were conserved across investigated genomes and homologs of *TaSHMT3A-1* were found in all investigated genomes **(B)**. Blackline, 1-to-1-mutual-best. Greenline, 1-to-its-best. Yellowline, 1-to-many. RBH, “reciprocal best hits”; SBH “single-side best hits”.

The micro-collinearity analysis helps to understand the replication or loss events of specific genes in the process of evolution or domestication; provides an opportunity to explore the inheritance and variation of genes in a local region, and can trace the origin history of a gene (Chen Y. et al., 2020). TGT was used to trace the origin history of the target gene under the guidance of the best matched collinear region. To explore the origin of *SHMT* genes in Triticeae species, 4 *SHMTs* of subgenomes A of wheat were used as query genes for micro-collinearity analysis; the results showed that homologs of *TaSHMT1A-1*, *TaSHMT2A-1*, and *TaSHMT4A-1* were found in the collinearity regions of *T. urartu*, *Ae. Tauschii*, subgenomes A and B of *T. dicoccoides* and subgenomes B and D of wheat (Supplementary Figure 1). This indicated that the evolution process of most *SHMTs* and their adjacent genes in the micro-collinearity regions of common wheat is relatively conservative, and it mainly enters common wheat through polyploidy. However, when *TaSHMT3A-1* was used as a query gene, the micro-collinearity relationship showed that no homolog of *TaSHMT3A-1* and its two neighboring genes was found in the collinearity region of *T. urartu*, and there were 27 genes inserted into the collinearity region of *T. urartu* (Figure 3A). The micro-collinearity relationship was further analyzed by removing the genome of *T. urartu* (Figure 3B). The results showed that *TaSHMT3A-1* and its homologs showed high similarity in the micro-collinearity regions of different genomes, suggesting that the corresponding *SHMT* and its two neighboring genes in *T. urartu* may have been lost. Some duplication events in the local region also occurred during the evolution of *T. urartu*, or a poor reference genome sequence of *T. urartu* caused this phenomenon.

Based on the fact that there was an *SHMT* gene, *TdSHMT7B-1*, which was on chromosome 7B of *T. dicoccoides*, while other analyzed Triticeae species did not contain the SHMT gene in the homologous group 7, and the number of exons and conserved motifs of this gene was less than that of other genes in the same subclass, *TdSHMT7B-1* was used as query gene for micro-collinearity analysis. However, there was no micro-collinearity of *TdSHMT7B-1* and its neighboring genes with other analyzed Triticeae species; furthermore, *TdSHMT7B-1* was a gene pair with *TRITD7Bv1G144890* of *Triticum turgidum*. The above results suggest that *SHMT* in tetraploid wheat may have experienced different evolution processes from other *SHMT* genes.

### Functional Diversification Analysis

DIVERGE v3.0 software was used to analyze the functional divergence of SHMT proteins in different subclasses (Table 3). The results showed that the θ value of Type -I ranged from 0.3337 to 0.6333, the degree of functional differentiation among subclasses fluctuated wildly, and the standard error was 0.06. The *P*-values of different subclasses were lower than 0.01, indicating that functional differentiation was caused by the change of evolution rate among subclasses (Gu et al., 2013; Xu et al., 2021). The Type -II analysis showed that θ ranged within 0.1584–0.3326 (Table 3), except class I a/I b. The differences among other subgroups reached a significant level (*P* < 0.01), indicating that there was functional differentiation caused by the constant evolution rate, but the change of corresponding amino acid characteristics among these subgroups meant that there were some changes of critical amino acid sites which caused the functional divergence among the above subclasses. The above results indicate that gene functional divergence of different classes (except class I a/I b) came from both changing of some critical amino acid sites and evolution rate.

**TABLE 3:** The result of Type-I and -II functional divergence.

Subgroup	I			II		
	MFE	MFE se	I :P	Theta-II	Theta SE	II :P
I a/I b	0.3337	0.0606	1.4905E-10**	0.1699	0.0660	0.0101
I a/II a	0.4038	0.0743	8.5050E-10**	0.1584	0.0565	0.0051**
I a/II b	0.4128	0.0670	6.8656E-13**	0.2122	0.0573	0.0002**
I b/II a	0.3833	0.0691	2.2837E-10**	0.1940	0.0509	0.0001**
I b/II b	0.6333	0.0694	0**	0.3326	0.0499	2.5331E-11**
II a/II b	0.4793	0.0757	4.0479E-13**	0.2130	0.0404	1.3256E-07**

MFE, model-free method. **Significance at p-values less than 0.01.

### Expression Patterns Analysis of *TaSHMTs*

The expression patterns of gene family members are helpful in predicting their potential biological functions. To elucidate the potential role of *TaSHMTs*, their expression patterns were studied by qRT-PCR. Expression patterns of *TaSHMTs* under two biotic stresses (powdery mildew pathogen and *F. graminearum*), abiotic stresses (PEG, NaCl, and 4°C), in different tissues (roots, stems, and leaves of the seedling stage) and two hormone treatments (Abscisic acid: ABA and H_2_O_2_) were analyzed. Because the sequence similarity of the three copy genes in different subgenomes of wheat is high, qRT-PCR primers could not effectively distinguish the three copy genes; therefore, the gene of subgenomes A was used to represent the relative expression of the three copy genes. The expression of *TaSHMTs* on chromosomes 4A, 4B, and 4D could not be detected in different tissues, so the relative expression data of *TaSHMTs* gene in homologous group 4 were not provided in this study.

**FIGURE 4:**
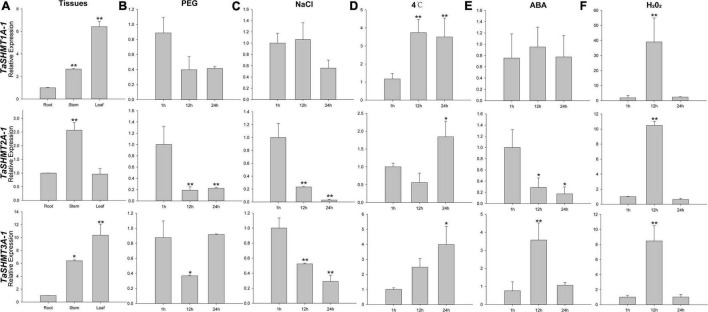
Relative expressions of three *TaSHMT* genes in different tissues, under different abiotic stresses and response to ABA and H_2_O_2_ treatment by qRT-PCR. Expression profiling of *TaSHMT* genes. Tissues were sampled from Sumai3 at the adult stage **(A)**. Fourteen -day -old seedling leaves were sampled after 1, 12, and 24 h under stress conditions comprising 20% PEG6000 **(B)**, 200 mM NaCl **(C)**, cold (4°C) **(D)**, 100 μmol H_2_O_2_ **(E)** and 100 μmol ABA **(F)**. Asterisks indicate significant differences (assessed using Duncan’s honestly significant difference test), *P < 0.05, **P < 0.01. All the raw data for qRT-PCR are listed in Supplementary Table 3.

The expression pattern of *TaSHMT* genes in different tissues, under various abiotic stresses and response to ABA and H_2_O_2_ treatments is shown in Figure 4. The expression of analyzed *TaSHMTs* in stem and leaf were higher than that in root (Figure 4A). With PEG and NaCl treatments, the relative expression of *TaSHMT2A-1* and *TaSHMT3A-1* was down-regulated, and the difference was significant, but the expression of *TaSHMT1A-1* did not change significantly (Figures 4B,C). Under the cold treatment, the relative expression of all *TaSHMT* genes was up-regulated (Figure 4D). With ABA treatment, the expression patterns of the three *TaSHMT* genes were different, the relative expression of *TaSHMT1A-1* did not change significantly at different time points (Figure 4E); *TaSHMT2A-1* was down-regulated, *TaSHMT3A-1* showed a rapid increase and reached peak levels at 12 h, then returned to the original level at 24 h (Figure 4E). The transcriptional responses of *TaSHMT* genes to H_2_O_2_ showed that *TaSHMT1A-1* (39-fold), *TaSHMT2A-1* (10-fold), and *TaSHMT3A-1* (8-fold) were highly upregulated at 12 h and then returned to the original level at 24 h (Figure 4F).

**FIGURE 5:**
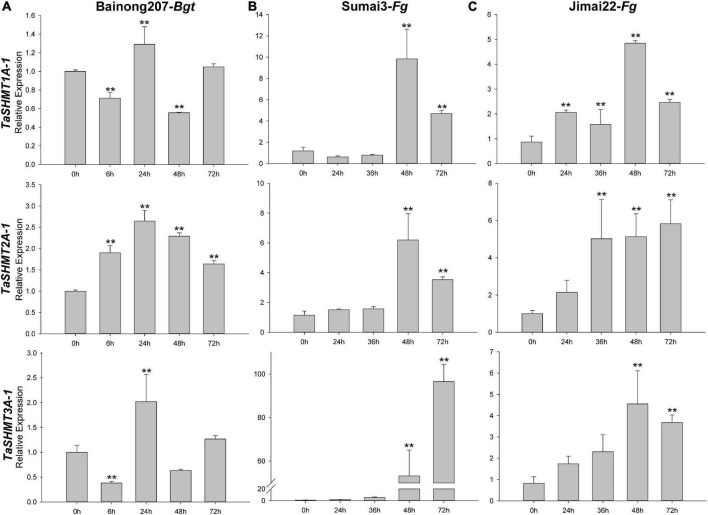
Relative expressions of three *TaSHMT* genes after *Bgt* inoculation and *F. graminearum* by qRT-PCR. Expression profiling of three *TaSHMT* genes in response to *Bgt* **(A)** and *F. graminearum* **(B–C)**. Data were normalized to the *TaTubulin* gene. The values are the means of three technical replicates of one biological experiment. Error bars indicate the standard error. Asterisks indicate significant differences (assessed using Duncan’s honestly significant difference test), **P < 0.01. *Bgt*, *Blumeria graminis* f. sp. *tritici*; *Fg*, *Fusarium graminearum.*

After *Bgt* inoculation, the relative expression levels of *TaSHMT1A-1* and *TaSHMT3A-1* were down-regulated at 6 h, then reached the expression peak at 24 h; the relative expression levels of *TaSHMT2A-1* were up-regulated at 6 h and then reached the expression peak at 24 h (Figure 5A); however, the absolute times of relative expression change were small. The expression patterns of *TaSHMTs* in the FHB resistant cultivar Sumai 3 and the susceptible cultivar Jimai 22 at different times after infection with *F. graminearum* were further analyzed by qRT-PCR. For Sumai 3 and Jimai 22, all the relative expressions of the three *TaSHMT1A-1*, *TaSHMT2A-1*, and *TaSHMT3A-1* were up-regulated in both materials (Figures 5B,C). Remarkably, the transcript levels of *TaSHMT3A-1* rapidly reached levels at 48 and 72 h were 46- and 101-fold higher than at 0 h in Sumai 3, respectively; furthermore, although the expression of *TaSHMT3A-1* in the susceptible material Jimai22 was also up-regulated, the levels that at 48 and 72 h were threefold higher than at 0 h. Therefore, we deduced that *TaSHMTs*, especially the *TaSHMT3A-1* might play an important role in the resistance response to FHB.

### Silencing of *TaSHMT3A-1* Compromises Fusarium Head Blight Resistance in Common Wheat Bainong207

**FIGURE 6:**
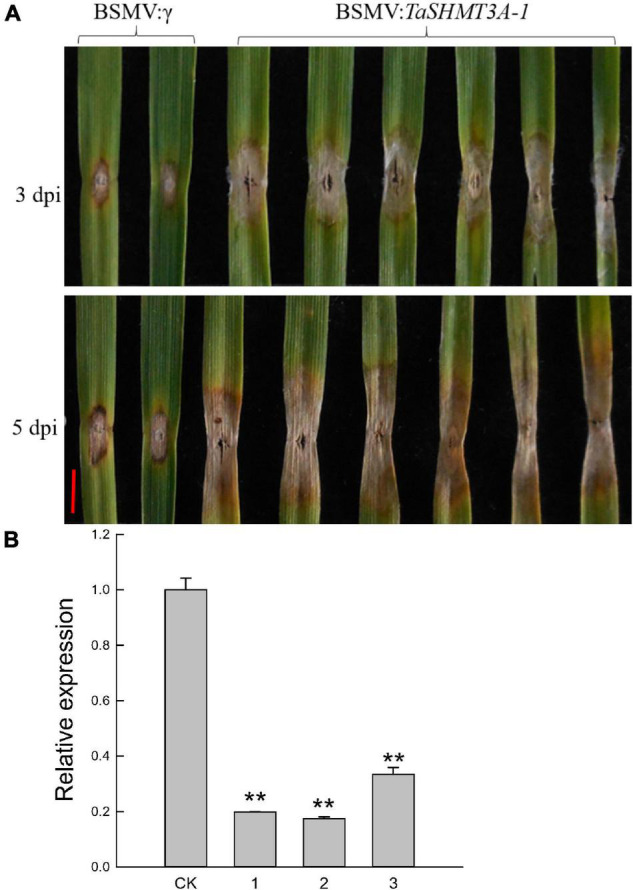
Functional analysis of *TaSHMT3A-1* by Barley stripe mosaic virus-based virus-induced gene silencing (BSMV-VIGS) in Bainong207. **(A)** BSMV: *TaSHMT3A-1* infected individual plants were inoculated with *F. graminearum*, and their leaves were photographed at 3 and 5 days post-inoculation (dpi). BSMV:γ were performed as a control. The experiment was repeated independently three times and the same results were obtained. Scale bar, 5 mm. **(B)** Expression of *TaSHMT3A-1* at 14 days in BSMV: *TaSHMT3A-1*-infected leaves was compared with that in BSMV:γ-infected controls of Bainong207. CK represents plants inoculated with BSMV:γ, and 1-3 represents plants inoculated with BSMV: *TaSHMT3A-1*. Asterisks show significant differences compared with the control (Duncan’s honestly significant difference test), **P < 0.01.

Previous studies have shown that the Jasmonic Acid signaling pathway is related to wheat FHB resistance (Xiang et al., 2011), and exogenous ABA treatment can increase wheat sensitivity to FHB (Qi et al., 2016). In this study, *TaSHMT3A-1* gene quickly responded to the induction of ABA and MeJA (Figure 5A and Supplementary Figure 2), and the relative expression of *TaSHMT3A-1* in the FHB resistant cultivar Sumai 3 was significantly increased by 101 times at 72 h. Therefore, *TaSHMT3A-1* was selected to further analyze its potential role in wheat resistance to FHB. The BSMV-VIGS system was used to further characterize the function of the *TaSHMT3A-1* gene in common wheat Bainong207. BSMV: *SHMT3A-1*, which carries a 249-bp *TaSHMT3A-1* fragment was used to induce target silencing. The fourth fully expanded leaves of BSMV: *TaSHMT3A-1*-infected plants were detached, followed by inoculation with a fresh *F. graminearum* spore suspension and RNA extraction. Leaves of the same age from BSMV:γ-infected plants were collected and inoculated with fresh *F. graminearum* as controls. Three and five days after infection, BSMV: *TaSHMT3A-1*-infected leaves were more susceptible to FHB than those BSMV:γ-infected individuals (Figure 6A). The expression levels of *TaSHMT3A-1* were significantly decreased, by 3–6-fold, as assessed by qRT-PCR (Figure 6B). Therefore, silencing the *TaSHMT3A-1* gene could increase the susceptibility of Bainong207 to FHB.

## Discussion

### Evolutionary Relationship of *SHMT* Genes in Triticeae Species

Serine hydroxymethyltransferase is involved in the reversible interconversion of Ser and Gly, participates in amination and decarboxylation reactions, and plays an important role in the cell-carbon metabolic pathway (Bauwe and Kolukisaoglu, 2003; Voll et al., 2006). Previous studies mainly focused on the function of *SHMT* genes under abiotic stresses such as light intensity, salt, and drought (Voll et al., 2006; Cui et al., 2019; Mishra et al., 2019); however, studies on the role of the *SHMT* gene family in biotic stresses and its evolutionary relationship in important crops at the genomic level have been limited. Wheat is a heterologous hexaploid composed of three subgenomes A, B, and D. Studies reveal that hybridization of *T. urartu* (AA) and an *Ae. speltoides*-related grass (BB) produced a tetraploid ancestor *T. turgidum* (AABB). After the second hybridization of *Ae. tauschii* (DD) and *T. turgidum*, the hexaploid ancestor (AABBDD) of wheat was formed (Shewry, 2009; Allaby et al., 2017). With the related species of wheat being sequenced, the evolutionary history of wheat has been relatively clear (Avni et al., 2017; Luo et al., 2017; IWGSC, 2018; Ling et al., 2018), and it is an important goal of future research to infer when some wheat gene subfamilies expanded and occurred during the evolutionary process (Schilling et al., 2020).

In this study, bioinformatics methods were used to comprehensively analyze the *SHMT* gene family in wheat, and to investigate its potential role in development and biotic and abiotic stresses. A total of 64 genes containing the complete conserved domain of SHMT (PF00464) were screened from the genomic data of six monocotyledons and four dicotyledons and divided into four subclasses. The number of *SHMT* genes identified from the whole genome of *H. vulgare* (HH), *T. urartu* (AA), *Ae. tauschii* (DD), *T. dicoccoides* (BBAA) and wheat (BBAADD) were 4, 3, 4, 9, and 12, respectively. The proportion of *SHMT* number among species was consistent with the proportion of the corresponding genomic multiples; the number of genes without copy genes was almost the same (Table 1). Gene replication is the most common mechanism for gene family extension (Cannon et al., 2004; Yu et al., 2020) and results in functional differentiation, which is critical for environmental adaptation and speciation (Conant and Wolfe, 2008). This study showed no tandem duplication or segmental replication in wheat *SHMT* gene. Meanwhile, macro-collinear and micro-collinear analysis showed that the SHMT gene of wheat was derived from a polyploidization process rather than self-replication (Figure 3 and Supplementary Figures 1, 3). Interestingly, there was a *TdSHMT7B-1* on chromosome 7B of *T. dicoccoides* and there was no gene pair in wheat and other related species analyzed in this study; however, there was a homologous gene *TRITD7Bv1G144890* on chromosome 7B of heterotetraploid species *T. turgidum*. We extended our focus to the incomplete domain SHMT of Triticeae species. The chromosomes 7B and 4B of wheat; 7A of *T. dicoccoides*; 4D of *Ae. tauschii*, and 4A and 7A of *T. urartu* all had one incomplete SHMT gene (Supplementary Table 2). Compared with the SHMT gene in the same subgroup, TdSHMT7B-1 lacked motifs 7, 9, and 17 (Figure 2B). Structural and conserved motif analysis revealed that motif, intron/exon loss or gain may occur during the evolution of Triticeae *SHMT* and similar events also appeared in *SHMT* of soybean (Lakhssassi et al., 2019). Some studies suggest that, in monocots, a special gene loss event occurred in class II a, and a gene duplication event in class I a compensated for this loss (Lakhssassi et al., 2019). However, this study does not support the above viewpoint. Instead, we speculate that the loss of *SHMT* gene did occur in class II a of monocotyledons, but there was no particular replication in class I a to make up for it. On the contrary, the loss or regression of *SHMT* gene occurred in class I a of monocotyledons during the process of evolution. This difference in genetic evolution may be because the effect of the circadian clock on daily growth rhythms is different between monocotyledons and dicotyledons (Campoli et al., 2012). However, this speculation needs further research.

### *TaSHMT3A-1* Positively Regulates Fusarium Head Blight Resistance

In the process of evolution, duplicated genes often undergo functional differentiation, leading to neofunctionalization, subfunctionalization or non-functionalization (Prince and Pickett, 2002). Compared with the traditional “paralog” and “ortholog,” “homolog” can more accurately and reasonably understand the relationship of genes in polyploid species (Chen Y. et al., 2020). To better understand the evolution of *SHMT*, Diverge3.0 was used to analyze the functional differentiation of the *SHMT* gene in each subclass. Type I differentiation represents the difference in gene evolution rate, and Type II differentiation represents the change in physical and chemical properties of amino acids (Yang et al., 2020). The results showed that the gene functional differences among the subgroups (except class I a/I b) came not only from changes of some key amino acid loci, but also from changes in evolution rate. The results of class I a/I b showed that the functional differentiation between the two subgroups was mainly Type I, and it was mainly determined by the sites conserved in one subclass and not conserved in the other subclass. Gene expansion provides conditions for the formation of new functions. The sequence of one gene remains relatively stable and the sequence of the other expanded gene changes, and functional differentiation occurs.

Since the sequence similarity of the three copy genes in different subgenomes of wheat is high (Pfeifer et al., 2014), we used the universal qRT-PCR primers to analyze the expression levels of corresponding three copy genes. The *SHMT* gene was shown to be closely related to photosynthesis, and with reduced activities of SHMT will usually show severe growth retardation (Heineke et al., 2001; Somerville, 2001). In the present study, the results showed that the expression level of *TaSHMTs* in stems and leaves were higher than in roots, which may be because *SHMT* plays an important role in plant one-carbon metabolism and photorespiration, and green tissues are important parts of photorespiration. The expressions of *TaSHMT2A-1 and TaSHMT3A-1* were down-regulated under the stress of PEG and NaCl and *TaSHMT1A-1* did not respond to these two treatments. These results are consistent with previous research results in diploid wheat *Triticum monococcum* (Bhuiyan et al., 2007), speculating that *SHMTs* also have similar functions as in *T. monococcum.* With ABA treatment, the expression levels of *TaSHMT2A-1* were down-regulated, and *TaSHMT3A-1* showed a rapid increase (Figure 4E). Furthermore, *TaSHMT1A-1*, *TaSHMT2A-1*, and *TaSHMT2A-1* significantly responded to H_2_O_2_ (Figure 4F). Previous studies showed that *AtSHMT1* could prevent cell death and reduce reactive oxygen species accumulation during salt stress (Zhou et al., 2012). Furthermore, *AtSHMT1* play a crucial role in plant abiotic stress tolerance and ABA-induced stomatal movements (Liu et al., 2019), suggesting *TaSHMT2A-1* and *TaSHMT3A-1* may be involved in H_2_O_2_ - and ABA-induced tolerance to abiotic stress.

Wheat FHB is a serious disease of wheat, which seriously affects the human food and animal feed security (Li et al., 2019; Su et al., 2019; Wang et al., 2020). In this study, we found that the expression of *TaSHMT3A-1* in the FHB resistant cultivar Sumai 3 was significantly increased by 101 times after infection by *F. graminearum* at 72 h (Figure 5B). When the *TaSHMT3A-1* was silenced in Bainong207, the results showed that silencing the *TaSHMT3A-1* gene could increase the susceptibility of Bainong207 to FHB. Given that the link of the transcriptional response of *TaSHMT3A-1* to *F. graminearum*, ABA, MeJA and the result of BSMV-VIGS (Figures 4E,F, 6 and Supplementary Figure 2), it is reasonable for us to conclude that *TaSHMT3A-1* can enhance wheat resistance to FHB. The previous study indicated that *Arabidopsis shmt1-1* mutants were more susceptible than control plants infection with biotrophic and necrotrophic pathogens (Moreno et al., 2005). The soybean cyst nematode (SCN) resistance major genetic locus Rhg4 encodes an SHMT protein (Kandoth et al., 2017; Lakhssassi et al., 2019). Based on the above research, we speculate that the *SHMT* gene may play a vital role in plant disease and insect resistance. In this study, the function of *TaSHMT3A-1* on FHB was verified through a leaf BSMV-VIGS assay. The effects and contribution of *TaSHMT3A-1* gene on wheat FHB need to be further verified through stable genetic transformation plants. Although the function of the wheat *TaSHMTs* needs to be elucidated, this study revealed that *TaSHMTs* might have diverged new functions during the course of evolution.

## Conclusion

In the present study, 64 *SHMT* members were identified from six monocotyledon and four dicotyledon species, and phylogenetic relationship analysis of *SHMT* members classified them into two main classes. The gene structure and motif composition analyses revealed that SHMTs kept relatively conserved within the same subclasses; however, *TdSHMT7B-1* on chromosome 7B of *T. dicoccoides* had a special gene structure and motifs. Combined with micro-collinearity analysis, *TdSHMT7B-1*, *TaSHMT3A-1* and corresponding homologs in Triticeae species may have experienced a special evolutionary process. The expression pattern showed that *TaSHMT3A-1* was responsive to some abiotic, and biotic stresses, and hormone treatments. Significantly, upon *F. graminearum* infection the expression of *TaSHMT3A-1* was highly upregulated in resistant cultivar Sumai3. More importantly, silencing of *TaSHMT3A-1* compromised FHB resistance in common wheat Bainong207. Our new findings suggest that *TaSHMT3A-1* gene in wheat plays an important role in resistance to FHB. This study provides a valuable reference for further functional study of these genes.

## Data Availability Statement

The original contributions presented in the study are included in the article/Supplementary Material, further inquiries can be directed to the corresponding author/s.

## Author Contributions

PH and JX conceived and designed the experiments, analyzed the data, and wrote the manuscript. PH, PS, JX, QW, YT, YR, and YY performed the experiments and collected the data. DL, HH, and CL revised the manuscript. All the authors read and approved the final manuscript.

## Conflict of Interest

The authors declare that the research was conducted in the absence of any commercial or financial relationships that could be construed as a potential conflict of interest.

## Publisher’s Note

All claims expressed in this article are solely those of the authors and do not necessarily represent those of their affiliated organizations, or those of the publisher, the editors and the reviewers. Any product that may be evaluated in this article, or claim that may be made by its manufacturer, is not guaranteed or endorsed by the publisher.

## Footnotes

1
http://smart.embl-heidelberg.de/

2
http://www.wheatgenome.org/

3
http://www.mbkbase.org/Tu/

4
http://plants.ensembl.org/index.html

5
http://pfam.xfam.org/

6
https://www.ncbi.nlm.nih.gov/Structure/cdd/wrpsb.cgi

7
https://www.evolgenius.info/

8
http://memesuite.org/tools/meme

9
http://wheat.cau.edu.cn/TGT/

## References

[B1] Allaby R. G. Stevens C. Lucas L. Maeda O. Fuller D. Q. (2017). Geographic mosaics and changing rates of cereal domestication. *Philos. Trans. R. Soc. Lond B. Biol. Sci.* 372:20160429. 10.1098/rstb.2016.0429 29061901 PMC5665816

[B2] Avni R. Nave M. Barad O. Baruch K. Twardziok S. O. Gundlach H. (2017). Wild emmer genome architecture and diversity elucidate wheat evolution and domestication. *Science* 357 93–97. 10.1126/science.aan0032 28684525

[B3] Bai G. Shaner G. (2004). Management and resistance in wheat and barley to fusarium head blight. *Annu. Rev. Phytopathol.* 42 135–161. 10.1146/annurev.phyto.42.040803.140340 15283663

[B4] Bauwe H. Kolukisaoglu U. (2003). Genetic manipulation of glycine decarboxylation. *J. Exp. Bot.* 54 1523–1535. 10.1093/jxb/erg171 12730263

[B5] Bhuiyan N. H. Liu W. Liu G. Selvaraj G. Wei Y. King J. (2007). Transcriptional regulation of genes involved in the pathways of biosynthesis and supply of methyl units in response to powdery mildew attack and abiotic stresses in wheat. *Plant Mol. Biol.* 64 305–318. 10.1007/s11103-007-9155-x 17406792

[B6] Campoli C. Shtaya M. Davis S. J. Von Korff M. (2012). Expression conservation within the circadian clock of a monocot: natural variation at barley Ppd-H1 affects circadian expression of flowering time genes, but not clock orthologs. *BMC Plant Biol.* 12:97. 10.1186/1471-2229-12-97 22720803 PMC3478166

[B7] Cannon S. B. Mitra A. Baumgarten A. Young N. D. May G. (2004). The roles of segmental and tandem gene duplication in the evolution of large gene families in *Arabidopsis thaliana*. *BMC Plant Biol.* 4:10. 10.1186/1471-2229-4-10 15171794 PMC446195

[B8] Chakraborty S. Newton A. C. (2011). Climate change, plant diseases and food security: an overview. *Plant Pathol.* 60 2–14.

[B9] Chen C. Chen H. Zhang Y. Thomas H. R. Frank M. H. He Y. (2020). TBtools: an integrative toolkit developed for interactive analyses of big biological data. *Mol. Plant* 13 1194–1202. 10.1016/j.molp.2020.06.009 32585190

[B10] Chen Y. Song W. Xie X. Wang Z. Guan P. Peng H. (2020). A collinearity-incorporating homology inference strategy for connecting emerging assemblies in the triticeae tribe as a pilot practice in the plant pangenomic era. *Mol. Plant* 13 1694–1708. 10.1016/j.molp.2020.09.019 32979565

[B11] Conant G. C. Wolfe K. H. (2008). Turning a hobby into a job: how duplicated genes find new functions. *Nat. Rev. Genet.* 9 938–950. 10.1038/nrg2482 19015656

[B12] Cui G. Zhao Y. Zhang J. Chao M. Xie K. Zhang C. (2019). Proteomic analysis of the similarities and differences of soil drought and polyethylene glycol stress responses in wheat (*Triticum aestivum* L.). *Plant Mol. Biol.* 100 391–410. 10.1007/s11103-019-00866-2 30953278

[B13] Dean R. Van Kan J. A. Pretorius Z. A. Hammond-Kosack K. E. Di Pietro A. Spanu P. D. (2012). The Top 10 fungal pathogens in molecular plant pathology. *Mol. Plant Pathol.* 13 414–430. 22471698 10.1111/j.1364-3703.2011.00783.x PMC6638784

[B14] Del Ponte E. M. Fernandes J. M. C. Pavan W. Baethgen W. E. (2009). A model-based assessment of the impacts of climate variability on Fusarium head blight seasonal risk in Southern Brazil. *J. Phytopathol.* 157 675–681.

[B15] El-Gebali S. Mistry J. Bateman A. Eddy S. R. Luciani A. Potter S. C. (2019). The Pfam protein families database in 2019. *Nucleic Acids Res.* 47 D427–D432. 10.1093/nar/gky995 30357350 PMC6324024

[B16] Engel N. Ewald R. Gupta K. J. Zrenner R. Hagemann M. Bauwe H. (2011). The presequence of *Arabidopsis* serine hydroxymethyltransferase SHM2 selectively prevents import into mesophyll mitochondria. *Plant Physiol.* 157 1711–1720. 10.1104/pp.111.184564 21976482 PMC3327202

[B17] Garcia-Canaveras J. C. Lancho O. Ducker G. S. Ghergurovich J. M. Xu X. C. Da Silva-Diz V. (2021). SHMT inhibition is effective and synergizes with methotrexate in T-cell acute lymphoblastic leukemia. *Leukemia* 35 377–388. 10.1038/s41375-020-0845-6 32382081 PMC7647950

[B18] Gu X. Zou Y. Su Z. Huang W. Zhou Z. Arendsee Z. (2013). An update of DIVERGE software for functional divergence analysis of protein family. *Mol. Biol. Evol.* 30 1713–1719. 10.1093/molbev/mst069 23589455

[B19] He Z. Zhang H. Gao S. Lercher M. J. Chen W. H. Hu S. (2016). Evolview v2: an online visualization and management tool for customized and annotated phylogenetic trees. *Nucleic Acids Res.* 44 W236–W241. 10.1093/nar/gkw370 27131786 PMC4987921

[B20] Heineke D. Bykova N. Gardestrom P. Bauwe H. (2001). Metabolic response of potato plants to an antisense reduction of the P-protein of glycine decarboxylase. *Planta* 212 880–887. 10.1007/s004250000460 11346965

[B21] Hu P. Liu J. Q. Xu J. F. Zhou C. Y. Cao S. Q. Zhou W. H. (2018). A malectin-like/leucine-rich repeat receptor protein kinase gene, RLK-V, regulates powdery mildew resistance in wheat. *Mol. Plant Pathol.* 19 2561–2574. 10.1111/mpp.12729 30030900 PMC6637979

[B22] IWGSC (2018). Shifting the limits in wheat research and breeding using a fully annotated reference genome. *Science* 361(6403):eaar7191. 10.1126/science.aar7191 30115783

[B23] Kandoth P. K. Liu S. Prenger E. Ludwig A. Lakhssassi N. Heinz R. (2017). Systematic mutagenesis of serine hydroxymethyltransferase reveals an essential role in nematode resistance. *Plant Physiol.* 175 1370–1380. 10.1104/pp.17.00553 28912378 PMC5664460

[B24] Kumar S. Stecher G. Li M. Knyaz C. Tamura K. (2018). MEGA X: molecular evolutionary genetics analysis across computing platforms. *Mol. Biol. Evol.* 35 1547–1549. 10.1093/molbev/msy096 29722887 PMC5967553

[B25] Lakhssassi N. Patil G. Piya S. Zhou Z. Baharlouei A. Kassem M. A. (2019). Genome reorganization of the GmSHMT gene family in soybean showed a lack of functional redundancy in resistance to soybean cyst nematode. *Sci. Rep.* 9:1506. 10.1038/s41598-018-37815-w 30728404 PMC6365578

[B26] Letunic I. Khedkar S. Bork P. (2021). SMART: recent updates, new developments and status in 2020. *Nucleic Acids Res.* 49 D458–D460. 10.1093/nar/gkaa937 33104802 PMC7778883

[B27] Li G. Q. Zhou J. Y. Jia H. Y. Gao Z. X. Fan M. Luo Y. J. (2019). Mutation of a histidine-rich calcium-binding-protein gene in wheat confers resistance to Fusarium head blight. *Nat. Genet.* 51 1106–1112. 10.1038/s41588-019-0426-7 31182810

[B28] Ling H. Q. Ma B. Shi X. Liu H. Dong L. Sun H. (2018). Genome sequence of the progenitor of wheat A subgenome *Triticum urartu*. *Nature* 557 424–428. 10.1038/s41586-018-0108-0 29743678 PMC6784869

[B29] Liu S. M. Kandoth P. K. Warren S. D. Yeckel G. Heinz R. Alden J. (2012). A soybean cyst nematode resistance gene points to a new mechanism of plant resistance to pathogens. *Nature* 492 256–260. 10.1038/nature11651 23235880

[B30] Liu Y. Mauve C. Lamothe-Sibold M. Guerard F. Glab N. Hodges M. (2019). Photorespiratory serine hydroxymethyltransferase 1 activity impacts abiotic stress tolerance and stomatal closure. *Plant Cell Environ.* 42 2567–2583. 10.1111/pce.13595 31134633

[B31] Lozano R. Hamblin M. T. Prochnik S. Jannink J. L. (2015). Identification and distribution of the NBS-LRR gene family in the Cassava genome. *BMC Genomics* 16:360. 10.1186/s12864-015-1554-9 25948536 PMC4422547

[B32] Lu S. Wang J. Chitsaz F. Derbyshire M. K. Geer R. C. Gonzales N. R. (2020). CDD/SPARCLE: the conserved domain database in 2020. *Nucleic Acids Res.* 48 D265–D268. 10.1093/nar/gkz991 31777944 PMC6943070

[B33] Luo M. C. Gu Y. Q. Puiu D. Wang H. Twardziok S. O. Deal K. R. (2017). Genome sequence of the progenitor of the wheat D genome *Aegilops tauschii*. *Nature* 551 498–502. 10.1038/nature24486 29143815 PMC7416625

[B34] McClung C. R. Hsu M. Painter J. E. Gagne J. M. Karlsberg S. D. Salome P. A. (2000). Integrated temporal regulation of the photorespiratory pathway. Circadian regulation of two *Arabidopsis* genes encoding serine hydroxymethyltransferase. *Plant Physiol.* 123 381–392. 10.1104/pp.123.1.381 10806255 PMC59012

[B35] McMullen M. Bergstrom G. De Wolf E. Dill-Macky R. Hershman D. Shaner G. (2012). A unified effort to fight an enemy of wheat and barley: fusarium head blight. *Plant Dis.* 96 1712–1728. 10.1094/PDIS-03-12-0291-FE 30727259

[B36] Mishra P. Jain A. Takabe T. Tanaka Y. Negi M. Singh N. (2019). Heterologous expression of serine hydroxymethyltransferase-3 from rice confers tolerance to salinity stress in *E. coli* and *Arabidopsis*. *Front. Plant Sci.* 10:217. 10.3389/fpls.2019.00217 30941150 PMC6433796

[B37] Moreno J. I. Martin R. Castresana C. (2005). *Arabidopsis* SHMT1, a serine hydroxymethyltransferase that functions in the photorespiratory pathway influences resistance to biotic and abiotic stress. *Plant J.* 41 451–463. 10.1111/j.1365-313X.2004.02311.x 15659103

[B38] Neuburger M. Rebeille F. Jourdain A. Nakamura S. Douce R. (1996). Mitochondria are a major site for folate and thymidylate synthesis in plants. *J. Biol. Chem.* 271 9466–9472. 10.1074/jbc.271.16.9466 8621617

[B39] Ohyanagi H. Tanaka T. Sakai H. Shigemoto Y. Yamaguchi K. Habara T. (2006). The rice annotation project database (RAP-DB): hub for *Oryza sativa* ssp. *japonica* genome information. *Nucleic Acids Res.* 34 D741–D744. 10.1093/nar/gkj094 16381971 PMC1347456

[B40] Pfeifer M. Kugler K. G. Sandve S. R. Zhan B. Rudi H. Hvidsten T. R. (2014). Genome interplay in the grain transcriptome of hexaploid bread wheat. *Science* 345:1250091. 10.1126/science.1250091 25035498

[B41] Prabhu V. Chatson K. B. Abrams G. D. King J. (1996). 13C nuclear magnetic resonance detection of interactions of serine hydroxymethyltransferase with C1-tetrahydrofolate synthase and glycine decarboxylase complex activities in *Arabidopsis*. *Plant Physiol.* 112 207–216. 10.1104/pp.112.1.207 8819325 PMC157939

[B42] Prince V. E. Pickett F. B. (2002). Splitting pairs: the diverging fates of duplicated genes. *Nat. Rev. Genet.* 3 827–837. 10.1038/nrg928 12415313

[B43] Qi P. F. Balcerzak M. Rocheleau H. Leung W. Wei Y. M. Zheng Y. L. (2016). Jasmonic acid and abscisic acid play important roles in host-pathogen interaction between *Fusarium graminearum* and wheat during the early stages of fusarium head blight. *Physiol. Mol. Plant Pathol.* 93 39–48.

[B44] Schilling S. Kennedy A. Pan S. Jermiin L. S. Melzer R. (2020). Genome-wide analysis of MIKC-type MADS-box genes in wheat: pervasive duplications, functional conservation and putative neofunctionalization. *New Phytol.* 225 511–529. 10.1111/nph.16122 31418861

[B45] Schirch V. Szebenyi D. M. (2005). Serine hydroxymethyltransferase revisited. *Curr. Opin. Chem. Biol.* 9 482–487. 16125438 10.1016/j.cbpa.2005.08.017

[B46] Shewry P. R. (2009). Wheat. *J. Exp. Bot.* 60 1537–1553. 19386614 10.1093/jxb/erp058

[B47] Somerville C. R. (2001). An early *Arabidopsis* demonstration. Resolving a few issues concerning photorespiration. *Plant Physiol.* 125 20–24. 11154287 10.1104/pp.125.1.20 PMC1539316

[B48] Su Z. Q. Bernardo A. Tian B. Chen H. Wang S. Ma H. X. (2019). A deletion mutation in TaHRC confers Fhb1 resistance to Fusarium head blight in wheat. *Nat. Genet.* 51 1099–1105. 10.1038/s41588-019-0425-8 31182809

[B49] Turner S. R. Ireland R. Morgan C. Rawsthorne S. (1992). Identification and localization of multiple forms of serine hydroxymethyltransferase in pea (*Pisum sativum*) and characterization of a cDNA encoding a mitochondrial isoform. *J. Biol. Chem.* 267 13528–13534. 1618853

[B50] Voll L. M. Jamai A. Renne P. Voll H. Mcclung C. R. Weber A. P. (2006). The photorespiratory *Arabidopsis* shm1 mutant is deficient in SHM1. *Plant Physiol.* 140 59–66. 10.1104/pp.105.071399 16339799 PMC1326031

[B51] Waditee-Sirisattha R. Sittipol D. Tanaka Y. Takabe T. (2012). Overexpression of serine hydroxymethyltransferase from halotolerant cyanobacterium in *Escherichia coli* results in increased accumulation of choline precursors and enhanced salinity tolerance. *FEMS Microbiol. Lett.* 333 46–53. 10.1111/j.1574-6968.2012.02597.x 22587350

[B52] Wang D. Liu H. Li S. Zhai G. Shao J. Tao Y. (2015). Characterization and molecular cloning of a serine hydroxymethyltransferase 1 (OsSHM1) in rice. *J. Integr. Plant Biol.* 57 745–756. 10.1111/jipb.12336 25641188

[B53] Wang H. W. Sun S. L. Ge W. Y. Zhao L. F. Hou B. Q. Wang K. (2020). Horizontal gene transfer of Fhb7 from fungus underlies *Fusarium* head blight resistance in wheat. *Science* 368 (6493):eaba5435. 10.1126/science.aba5435 32273397

[B54] Wang X. Cao A. Yu C. Wang D. Wang X. Chen P. (2010). Establishment of an effective virus induced gene silencing system with BSMV in *Haynaldia villosa*. *Mol. Biol. Rep.* 37 967–972. 10.1007/s11033-009-9766-1 19714483

[B55] Xiang Y. Song M. Wei Z. Tong J. Zhang L. Xiao L. (2011). A jacalin-related lectin-like gene in wheat is a component of the plant defence system. *J. Exp. Bot.* 62 5471–5483. 10.1093/jxb/err226 21862481 PMC3223046

[B56] Xie T. Chen C. Li C. Liu J. Liu C. He Y. (2018). Genome-wide investigation of WRKY gene family in pineapple: evolution and expression profiles during development and stress. *BMC Genomics* 19:490. 10.1186/s12864-018-4880-x 29940851 PMC6019807

[B57] Xing L. Hu P. Liu J. Witek K. Zhou S. Xu J. (2018). Pm21 from *Haynaldia villosa* encodes a CC-NBS-LRR protein conferring powdery mildew resistance in wheat. *Mol. Plant* 11 874–878. 10.1016/j.molp.2018.02.013 29567451

[B58] Xu J. Hu P. Tao Y. Song P. Gao H. Guan Y. (2021). Genome-wide identification and characterization of the Lateral Organ Boundaries Domain (LBD) gene family in polyploid wheat and related species. *PeerJ* 9:e11811. 10.7717/peerj.11811 34447619 PMC8364319

[B59] Yang J. F. Chen M. X. Zhang J. H. Hao G. F. Yang G. F. (2020). Genome-wide phylogenetic and structural analysis reveals the molecular evolution of the ABA receptor gene family. *J. Exp. Bot.* 71 1322–1336. 10.1093/jxb/erz511 31740933

[B60] Yu J. Xie Q. Li C. Dong Y. Zhu S. Chen J. (2020). Comprehensive characterization and gene expression patterns of LBD gene family in *Gossypium*. *Planta* 251:81. 10.1007/s00425-020-03364-8 32185507

[B61] Zhang X. Halder J. White R. P. Hughes D. J. Ye Z. Wang C. (2014). Climate change increases risk of fusarium ear blight on wheat in central China. *Ann. Appl. Biol.* 164 384–395.

[B62] Zhang Y. Sun K. H. Sandoval F. J. Santiago K. Roje S. (2010). One-carbon metabolism in plants: characterization of a plastid serine hydroxymethyltransferase. *Biochem. J.* 430 97–105. 10.1042/BJ20100566 20518745

[B63] Zhou H. Zhao J. Yang Y. Chen C. Liu Y. Jin X. (2012). Ubiquitin-specific protease16 modulates salt tolerance in *Arabidopsis* by regulating Na(+)/H(+) antiport activity and serine hydroxymethyltransferase stability. *Plant Cell* 24 5106–5122. 10.1105/tpc.112.106393 23232097 PMC3556978

