# Supplementary material for: Genome-Wide Analysis of Serine Hydroxymethyltransferase Genes in Triticeae Species Reveals That TaSHMT3A-1 Regulates Fusarium Head Blight Resistance in Wheat
Source: Front Plant Sci. 2022 Feb 10;13:847087. doi: 10.3389/fpls.2022.847087 (PMC8866830; doi:10.3389/fpls.2022.847087)
Supplement: Supplementary file 1 [file Data_Sheet_1.docx]

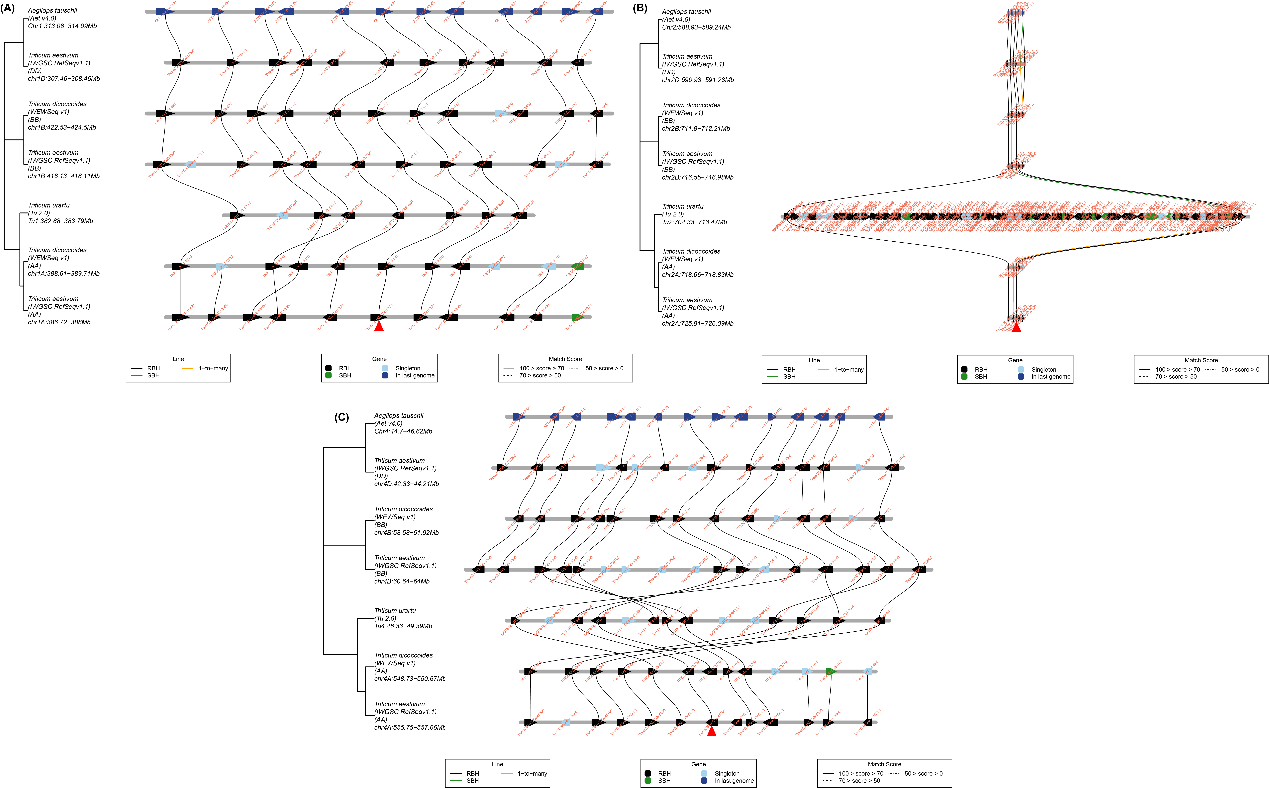


**Supplementary Figure 1. Micro-collinearity analysis by TGT to track the evolutionary history of *TaSHMT* homologs.** (**A-C**) *TaSHMT1A-1, TaSHMT2A-1, TaSHMT3A-1* was used as query genes, respectively. The micro-collinearity relationship showed that homologs of the analyzed *TaSHMTs* were found in all investigated genomes. Blackline, 1-to-1-mutual-best. Greenline, 1-to-its-best. Yellowline, 1-to-many. Abbreviations: RBH, ‘‘reciprocal best hits’’; SBH ‘‘single-side best hits’’.


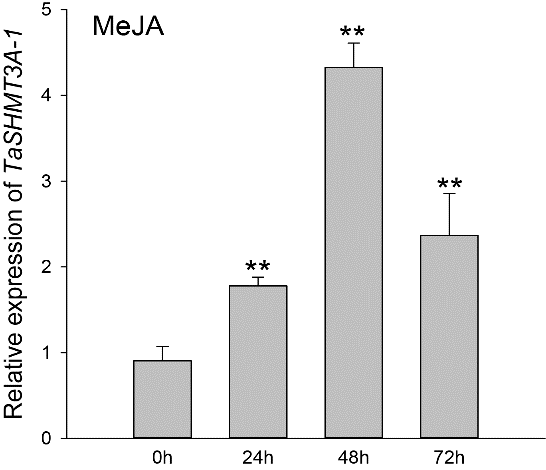


**Supplementary Figure 2.** **Relative expressions of *TaSHMT3A-1* response to MeJA treatment by qRT-PCR.** Expression profiling of *TaSHMT* genes. 14-day -old seedling leaves were sampled after 0, 24, 48, and 72 h post-treatment with 100 μmol MeJA. Data were normalized to the *TaTubulin* gene. Asterisks indicate significant differences (assessed using Duncan’s honestly significant difference test), * P < 0.05, ** P < 0.01. Abbreviations: MeJA, methyl jasmonate.

**
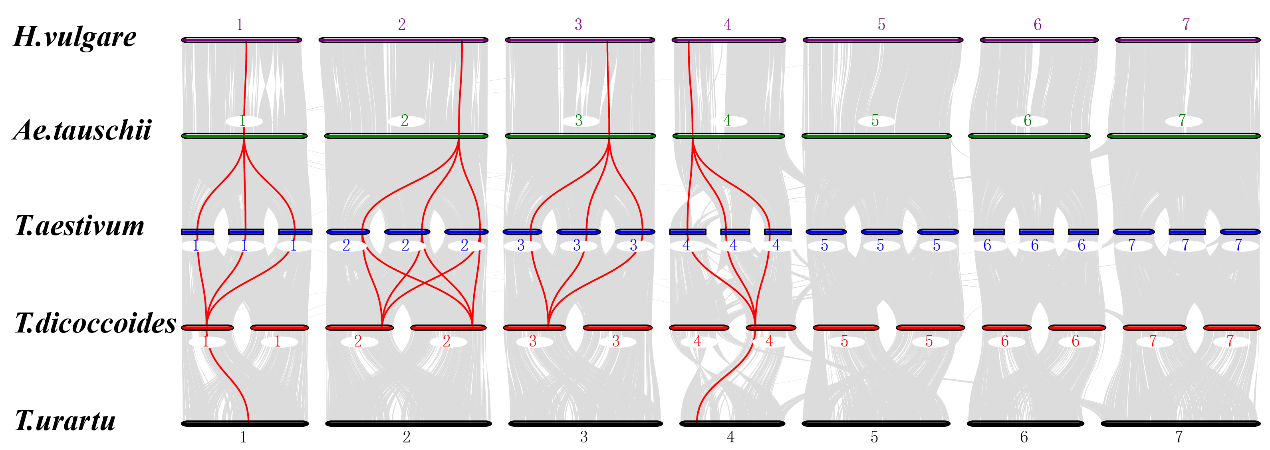
**

**Supplementary Figure 3. Macro-collinearity analysis of *SHMT* genes among Triticeae species.** Cascaded profile of macro-collinearity of different species constructed using TBtools. Collinearity relationships were shown between two adjacent species. Gray lines in the background indicate the collinear blocks within different genomes and the red lines highlight the syntenic *SHMT* gene pairs.
