# Supplementary material for: Genome-Wide Analysis of Serine Hydroxymethyltransferase Genes in Triticeae Species Reveals That TaSHMT3A-1 Regulates Fusarium Head Blight Resistance in Wheat
Source: Front Plant Sci. 2022 Feb 10;13:847087. doi: 10.3389/fpls.2022.847087 (PMC8866830; doi:10.3389/fpls.2022.847087)
Supplement: Supplementary file 2 [file Table_1.DOCX]

**Supplementary Table 1. Primer list.**

| Name | Sequence | Used for |
| --- | --- | --- |
| TaSHMT1A-1FQ | 5’- GCTGTGGTCATCTGCTTGAA | qRT-PCR |
| TaSHMT1A-1RQ | 5’- TGTCTGACACCAGGAATCCA |  |
| TaSHMT2A-1FQ | 5’- CGGCTACATCGACTACGACA | qRT-PCR |
| TaSHMT2A-1RQ | 5’- CCACATCGCAGAACTCAAAA |  |
| TaSHMT3A-1FQ | 5’- ATGTCCAGCCCTACTCATGC | qRT-PCR and Gene silencing efficiency analysis |
| TaSHMT3A-1RQ | 5’- GCCAGTCTGTGGATTCACCT |  |
| TaSHMT4A-1FQ | 5’- GTTCACCTGCCAACTTCCAT | qRT-PCR |
| TaSHMT4A-1RQ | 5’- AGCCAGTGCTTTCATCCAGT |  |
| Ta-Tubulin-F | 5’ATCTCCAACTCCACCAGTGTCG | qRT-PCR |
| Ta-Tubulin-R | 5’- TCATCGCCCTCATCACCGTC |  |
| VIGS-TaSHMT3A-1-F | 5’-TAGCTGAGCGGCCGCCCCG  GGTTGGGTGGAAATCCATTGC | Construct BSMV: *TaSHMT3A-1* vector |
| VIGS-TaSHMT3A-1-R | 5’- TAGCTGATTAATTAACCCG  GGATGTCCAGCCCTACTCATG |  |
